# The Combined Antioxidant Effects of N-Acetylcysteine, Vitamin D3, and Glutathione from the Intestinal–Neuronal In Vitro Model

**DOI:** 10.3390/foods13050774

**Published:** 2024-03-01

**Authors:** Simone Mulè, Sara Ferrari, Giorgia Rosso, Arianna Brovero, Mattia Botta, Alessia Congiusta, Rebecca Galla, Claudio Molinari, Francesca Uberti

**Affiliations:** 1 Laboratory of Physiology, Department for Sustainable Development and Ecological Transition, University of Piemonte Orientale, UPO, 13100 Vercelli, Italy; simone.mule@uniupo.it (S.M.); mattiabotta23@gmail.com (M.B.);; 2 Department of Clinical and Biological Sciences, University of Turin, 10043 Turin, Italy; 3 Noivita S.r.l.s., Spin Off of University of Piemonte Orientale, Via Solaroli 17, 28100 Novara, Italy

**Keywords:** oxidative stress, brain ageing, oral absorption, bioavailability, food supplement, antioxidant defence

## Abstract

Chronic oxidative stress has been consistently linked to age-related diseases, conditions, and degenerative syndromes. Specifically, the brain is the organ that significantly contributes to declining quality of life in ageing. Since the body cannot completely counteract the detrimental effects of oxidative stress, nutraceuticals’ antioxidant properties have received significant attention in recent years. This study assesses the potential health benefits of a novel combination of glutathione, vitamin D3, and N-acetylcysteine. To examine the combination’s absorption and biodistribution and confirm that it has no harmful effects, the bioavailability of the mixture was first evaluated in a 3D model that mimicked the intestinal barrier. Further analyses on the blood–brain barrier was conducted to determine the antioxidant effects of the combination in the nervous system. The results show that the combination reaches the target and successfully crosses the blood–brain and intestinal barriers, demonstrating enhanced advantages on the neurological system, such as a reduction (about 10.5%) in inflammation and enhancement in cell myelination (about 20.4%) and brain tropism (about 18.1%) compared to the control. The results support the cooperative effect of N-acetylcysteine, vitamin D3, and glutathione to achieve multiple health benefits, outlining the possibility of an alternative nutraceutical approach.

## 1. Introduction

The increase in longevity observed in recent years is, unfortunately, associated with an increase in age-related disorders. This results in an increasing burden on social and health systems. As the most complex organ in the human body, the brain is metabolically active and consumes nearly a quarter of the body’s total glucose and oxygen. This activity places the brain at the centre of the ageing process. The elevated oxygen consumption can result in heightened generation of reactive oxygen species (ROS) [1]. In the ageing brain, mitochondrial function is reduced, and damaged mitochondria cause ROS accumulation [2]. To mitigate the detrimental consequences of ROS formation, the human body has a sophisticated system of naturally occurring enzymatic and non-enzymatic antioxidant defences [3]. Despite the significant antioxidant and repair mechanisms developed by biological systems, oxidative damage is an unavoidable byproduct of aerobic living. Degenerative processes, illnesses, and syndromes have recently been linked to oxidative stress [4,5]. On the other hand, ROSs may function as intracellular signalling molecules when tightly controlled, playing a crucial role in complex cellular processes, including controlling blood pressure, cognitive abilities, and immunological responses [6]. In this context, in recent years, the main effect studied in nutraceuticals is the protection of cells from oxidative damage, which can be achieved by eliminating ROS and free radicals or trapping them [7,8]. Nutraceuticals exert their effects throughout the body by engaging with various targets in different organs, showcasing their activity at a systemic level [9,10]. There are many possibilities for dietary supplements, and it is important to objectively weigh the benefits and drawbacks of each option based on individual medical conditions. Generally, foods’ ability to exert anti-inflammatory, anti-hypertensive, anti-platelet, and antioxidant properties can be attributed to their polyphenol concentration [11]. In general, various foods and drinks, such as vegetables, fruits, chocolate, grain cereals, EVO olive oil, eggs, meat, legumes, nuts, red wine, and tea, all contain antioxidants at variable levels [12]. For example, a widely known natural antioxidant is N-acetylcysteine (NAC), whose primary role concerns its antioxidant and anti-inflammatory activity, which promotes the maintenance of a cellular redox balance. As a result, its therapeutic potential extends to several disorders whose aetiology and course are linked to oxidative stress [13,14]. In several cellular systems, NAC could promote beneficial effects that support the vital functions of the cells; more specifically, one of these effects is the stimulation of intracellular glutathione (GSH) production, which is recognized as the body’s primary antioxidant, since it plays a crucial role in safeguarding cells against oxidative stress and ensuring the maintenance of the redox state [15]. Nevertheless, NAC supplementation is contingent on the body’s capacity to produce glutathione from readily available basic materials. This capacity declines with advancing age and in specific medical conditions, particularly liver impairment [16]. The increasing curiosity in understanding the beneficial effects of NAC stems not only from its powerful cellular bio-protective action but also from its pharmacokinetic characteristics, which include safety, absorption, bioavailability, and cost-effectiveness [13,14]. Furthermore, another compound that is already known to have antioxidant activity is vitamin D3 (VitD3); specifically, concentrations of VitD3 are particularly crucial for preventing inflammation, eliminating parasites and microorganisms that have invaded the body, reducing oxidative stress after daily exposure to toxins, and slowing down the ageing process [17]. Indeed, VitD3 is commonly utilized as an antioxidant therapy to prevent or mitigate the imbalance between oxidant and antioxidant species [18]. VitD3 also promotes healthy mitochondrial function, which helps manage cellular oxidation and reduction processes that, when dysregulated, can result in increased ageing, neurological disorders, abnormal cell growth, and cell death [18]. Glutathione (Glut) is recognised to have effects like preserving redox balance, decreasing oxidative stress, enhancing metabolic detoxification, and regulating immune system function, which is especially relevant in the pathophysiology of various diseases [19]. Glutathione (Glut) is a key player in vital physiological processes. Nevertheless, it is crucial to remember that the benefits of NAC, VitD3, and Glut are typically linked to a specific biological process. Furthermore, it has been established that oral NAC has a poor absolute bioavailability, ranging from 6 to 10%, most likely due to substantial first-pass metabolism in the liver and gut wall [20]. NAC enters the systemic circulation via gastric or other intravenous routes; for it to exit the blood vessels, N-deacetylation or carrier-mediated active transport is required; however, no such method has been documented for NAC [21]. Therefore, it is necessary to promote research into methods for increasing the bioavailability and efficacy of NAC at its target sites. Numerous studies recommend concentrating on the nutraceuticals’ cooperative mechanism, looking into the interactions between herbal products and prescription drugs or biochemical components, and assessing different approaches to increase the health benefits of NAC [22,23]. Accordingly, this study aimed to evaluate a novel formulation containing NAC, VitD3, and Glut to obtain multiple health benefits due to their cooperative effects to explore a substitute for α-Lipoic Acid (LA), which is well known to provide neuroprotection in several in vitro models [24]. This was achieved by analysing how the combination of NAC, VitD3, and glutathione could improve the effectiveness of NAC. Considering the lack of data on the biological activity of these three active ingredients, a more in-depth investigation was needed. Consequently, the primary goal of this investigation was to evaluate uptake and biodistribution through major target organs using in vitro models in which the antioxidant machinery is malfunctioning. Furthermore, the specific biological activity and antioxidant capacity of NAC, combined with those of VitD3 and glutathione, were determined at each stage of the in vitro-simulated biodistribution process by evaluating gastrointestinal absorption, hepatic metabolism, and endogenous antioxidant activity in a peripheral/central nervous system (PNS/CNS) model.

## 2. Materials and Methods

### 2.1. Reagent Preparations

An investigation was conducted to determine whether NAC, VitD3, and Glut (all manufactured by Merck Life Science, Rome, Italy) could stimulate first-pass hepatic metabolism after crossing the intestinal barrier to reach the central and peripheral nervous systems. These substances were evaluated both individually and in combination. The concentrations utilised for all substances examined were obtained from the literature [25,26,27] and confirmed through dose–response experiments; these tests determined that the most effective concentrations were 12 mM NAC, 100 nM VitD3, and 5 mM Glut. The positive control employed in this study is LA 50 µM (Merck Life Science, Rome, Italy), chosen due to its proven effects on pain modulation pathways [26]. Every substance that underwent testing was prepared in Dulbecco’s Modified Eagle’s Medium (DMEM), obtained from Merck Life Science in Rome, Italy. It did not contain phenol red and was supplemented with 1% penicillin–streptomycin, 0.5% foetal bovine serum (FBS), and 2 mM L-glutamine (all from Merck Life Science in Rome, Italy).

### 2.2. Cell Cultures

The CaCo-2 cell line, derived from human Caucasian colon adenocarcinoma from the American Type Culture Collection (ATCC, Manassas, VA, USA), was cultured in Dulbecco’s Modified Eagle’s Medium Advance (DMEM-Adv, Thermo Fisher Scientific, Rodano, MI, Italy) supplemented with 10% FBS, 2 mM L-glutamine, and 1% penicillin–streptomycin at 37 °C in an incubator [28]. The EMA and FDA approve this cell line for experimental models that predict oral drug absorption, metabolism, and bioavailability [29,30]. Paracellular permeability and transport properties were balanced using cells at passage numbers 26–32 [28]. An MTT-based In Vitro Toxicology Assay Kit (MTT, Merck Life Science, Rome, Italy) was used to analyse cell viability at 1 × 10^4^ cells in 96-well plates. A total of 2 × 10^4^ cells were placed in a 24-well plate on 6.5 mm Transwell^®^ inserts with a 0.4 μm pore polycarbonate membrane to conduct an absorption investigation. The cells were washed before being stimulated, and they were then cultured in an incubator for eight hours in DMEM-Adv red-phenol-free media. The medium was enriched with 0.5% foetal bovine serum (FBS), 2 mM L-glutamine, and 1% penicillin–streptomycin [28].

The ATCC supplied the human epithelial hepatocellular carcinoma (HepG2) cells, which were incubated at 37 °C and 5% CO_2_ in DMEM-Adv supplemented with 10% FBS, 2 mM L-glutamine, and 1% penicillin–streptomycin [31]. This cell line is often used for hepatic metabolism studies when passage ranges are between 90 and 95 [32]. After achieving 80–90% confluence, the cells were cultured in a variety of methods by a variety of experimental protocols: 1 × 10^6^ cells were plated on a 6-well plate to study the inflammatory marker and the intracellular pathways by ELISA kit. A total of 2 × 10^5^ cells were plated on Transwell^®^ system to perform the LA quantification and permeability assay by 0.04% fluorescein (Merck Life Science, Rome, Italy). A total of 2 ×10^4^ cells were plated in 96-well plates for the analysis of cell viability by MTT test (Merck Life Science, Rome, Italy) [33].

HUVEC, a human umbilical vein endothelial cell line obtained from ATCC, was cultured in a flask coated with 0.1% gelatine and Endothelial Growth Medium-2 (EGM-2). EGM-2 (Lonza, Basel, Switzerland) was supplemented with the following substances: 0.4% vascular endothelial growth factor (VEGF), 0.4% human basic fibroblast growth factor (hFGF-B), 0.1% recombinant analogue of human insulin-like growth factor-I (R3-IGF-1), 0.1% ascorbic acid, 0.1% human epidermal growth factor (hEGF), and 0.1% Gentamicin Sulphate-Amphotericin (GA-1000) and 0.1% heparin [34]. A polyester membrane with a hole size of 0.4 μm was used to plate 1 × 10^5^ HUVEC cells/cm2 in the apical compartment of 6.5 mm Transwells^®^ to generate an in vitro blood–brain barrier (BBB).

CCF-STTG1 cells (ATCC) from a 68-year-old astrocytoma patient’s brain were cultured in Roswell Park Memorial Institute medium (RPMI, Merck Life Science, Rome, Italy) with 10% FBS, 2 mM Hepes, 2 mM L-Glutamine, and 1% penicillin–streptomycin in an incubator at 37 °C with 5% CO_2_ [35]. The cell growth was periodically monitored, and the culture medium was replaced every 48 h. The cells were employed for experimental objectives once their confluence level increased to 75% and 85%. This was accomplished within three passes of their procurement from the supplier and no later than three months later [35]. To generate an in vitro blood–brain barrier (BBB), 4 × 10^4^ cells/cm^2^ of cells were plated on the basolateral side of the reversed Transwells^®^ insert. Simultaneously, 1 × 10^4^ CCF-STTG1 cells were seeded into a 96-well plate for MTT analysis of cell viability, and 1 × 10^6^ cells were seeded into a 6-well plate for ELISA Kit analysis of tumour necrosis factor (TNFα) and interleukin 1b (IL1b).

Schwann RSC-96 cells (ATCC) derived from rats were cultured in DMEM-Adv containing 10% FBS, 2 mM L-glutamine, and 1% penicillin–streptomycin at 37 °C with 5% CO_2_. The cells were subcultured two–three times per week to obtain 10 to 15 passages, which were utilised in experiments to establish a co-culture system with PC12 cells (PNS in vitro model) [36,37]. This cell line is extensively utilised in research about the peripheral nervous system due to its ability to promote in vitro axon regeneration [37,38]. Rat neuronal PC12 (ATCC) cells were co-cultured with this cell line in RPMI supplemented with 5% FBS, 10% horse serum (HS; Merck Life Science, Rome, Italy), and 2 mM glutamine. Cells between passages 3 and 13 were utilised for investigations; the cultures were maintained at 37 °C with 5% CO_2_ at a sub-confluence of 70% to 80% [39]. PC12 cells are commonly utilised neuronal cell lines in in vitro screening for compounds with potential neuroprotective properties [40]. To generate an in vitro model of the peripheral nervous system (PNS), a co-culture system was employed to plate 4 × 10^6^ RSC96 cells and 1 × 10^5^ PC12 cells, thereby simulating the environment of the PNS [38].

### 2.3. Experimental Protocol

The experiments were organised in different phases corresponding to the specific cell types to explore the effects of NAC, VitD3, and Glut alone and combined. The CaCo-2 cells were utilised in the initial stage to rule out the cytotoxic effects of the substances under investigation in a time-dependent and dose–response study from 1 to 6 h. Glut concentrations varied from 2 to 20 mM [27], while NAC concentrations ranged from 4 to 14 mM [25], and VitD3 concentrations ranged from 1 to 100 nM [26]. In particular, the better concentration of VitD3 is 100 nM and Glut 5 mM, confirming the data reported in the literature [26,41], and NAC induced a greater effect with 12 mM, which is the reason to maintain these concentrations on all successive experiments comparing the results to LA 50 µM. A Transwell^®^ system was utilised to examine the effects of the chosen concentrations of NAC, VitD3, and Glut on an in vitro intestinal barrier. The effects of NAC 12 mM alone and in combination with VitD3 100 nM and Glut 5 mM were evaluated, and intestinal barrier integrity and cell viability were assessed within 6 h of stimulation [28].

In addition, the permeability and absorption rates were determined by evaluating the apparent permeability coefficient (Papp) and the total amount of NAC that crosses the intestinal barrier.

In the second phase, the basolateral environments of the intestinal barrier were successfully used to explore the hepatic metabolism for 24 h [42], using HepG2 cells analysing cell viability, the main intracellular pathways are involved in inflammation response and hepatic homeostasis.

In the third phase, the experiments examined the biological effect of NAC 12 mM, VitD3 100 nM, and Glut 5 mM alone and combined, on both CNS and the PNS in vitro models; they were stimulated for 24 h with the hepatic metabolise medium [26], studying the permeability in the blood–brain barrier (BBB) in vitro model, the cell viability, and the anti-inflammatory properties on neuronal cells. This enabled an analysis of the effects compared to those of LA 50 µM. At the same time, the effects on the PNS in vitro model using the RSC96/PC12 co-culture were investigated by analysing the molecular pathways that were also involved, including LA 50 µM.

**Figure 1:**
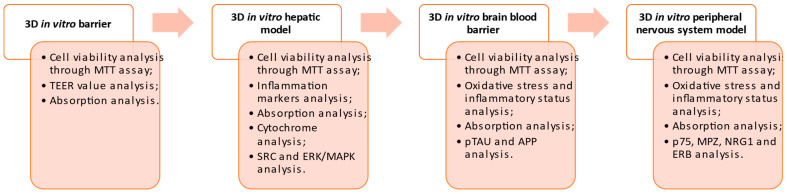
A schematic diagram of the experimental protocol is subdivided into phases, including the specific method used in each one.

The experimental plan used to carry out this study has been summarized in the diagram reported below (Figure 1)

### 2.4. In Vitro Model of the Intestinal Barrier

To estimate the absorption, metabolism, and bioavailability of several drugs following oral ingestion by humans, the EMA and FDA authorised a standard methodology published in the literature [29,30]. They recreated the in vitro intestinal barrier model using the Transwell^®^ technology [28]. The trans-epithelial electrical resistance (TEER) of CaCo-2 cells, which were cultured in a complete medium for 21 days before the simulations [38], was assessed using EVOM3 coupled with STX2 chopstick electrodes (World Precision Instruments, Sarasota, FL, USA). This monitoring allowed for the evaluation of mature intestinal epithelial formation and the occurrence of a proper paracellular mechanism around day 21, as indicated by TEER values of 400 Ωcm^2^ [43]. On the apical side, the media were adjusted to pH 6.5; this is the lumen pH of the small intestine before stimulation. On the basolateral side, where this was indicative of blood, the pH was 7.4 [28,44]. The TEER readings were examined at each stimulation interval to rule out any apical side integrity degradation. The Papp (cm/s) analysis [45], which measures permeability, was performed on the cells after they had been treated with all agents for one–six hours. The formula for this assay is as follows:

Papp = dQ/dt ⇥ 1/m0 ⇥ 1/A ⇥ V Donor

where dQ represents the quantity of substance that is being transported, measured in nanomoles (nmol) or micrograms (μg); dt represents the duration of the incubation period, measured in seconds (s); m0 represents the initial amount of substrate that is applied to the donor compartment, measured in nanomoles (nmol) or micrograms (μg); A is the measurement of the outside area of the Transwell^®^ membrane (cm^2^); VDonor is the amount of liquid in the donor compartment (cm^3^).

### 2.5. In Vitro Hepatic Environment

A proper maturation time of 3.5 × 10^4^ HepG2 cells was determined by TEER with EVOM3 coupled with STX2 chopstick electrodes (World Precision Instruments, Sarasota, FL, USA), until the cells reached a threshold of 486 Ωcm^2^ [46]. The cells were plated in the upper chamber of Transwell^®^ culture inserts with a pore size of 0.4 µm. Following the stimulation of the apical monolayer of HepG2 cells with basolateral intestinal medium, the basolateral media was collected to treat the BBB model and the co-culture RSC96/PC12.

### 2.6. BBB-CNS Axis

A BBB was generated by coculturing CCF-STTG1 astrocytic-like cells with HUVEC cells using procedures described in the literature [26]. A total of 4 × 10^4^ CCF-STTG1 cells/cm^2^ were seeded onto the basolateral surface of inverted 6.5 mm Transwells^®^ utilising a polyester membrane with a pore size of 0.4 μm (Corning Costar, Corning, NY, USA). The cells were then allowed to adhere for 4 hours. Following this, Transwell^®^ inserts were positioned in the conventional orientation, and the cells were allowed to proliferate for 48 h. Following this, an apical compartment was seeded with 1 × 10^5^ HUVEC cells/cm^2^. After that, the inserts were transferred to a 24-well plate. Permeability tests were conducted on the Transwell^®^ inserts after 7 days of culture, lasting from 15 to 1440 min [47]. The basolateral hepatic media triggered an intracellular route.

### 2.7. PNS In Vitro Model

According to the literature [38], it is essential to have the interaction between RSC96 and PC12 cell lines to establish the peripheral nerve environment. In summary, 1 mL of a solution comprising 80% *v*/*v* Type I rat tail collagen (2 mg/mL in 0.6% acetic acid, Thermo Fischer, Milan, Italy), 10% *v*/*v* Minimum Essential Medium (MEM, Merck Life Science, Milan, Italy), 5.8% *v*/*v* neutralising solution (Biosystems, Monza, Italy), and 4.2% Schwann cell suspension (4 × 10^6^ RSC96 cells per 1 mL gel) was placed inside a rectangular scaffold measuring 16.4 mm by 6.5 mm by 5 mm. Following gel solidification, the gel was immersed in a 10 mL DMEM solution and incubated for 24 h at 37 °C with 5% CO_2_ concentration. To stabilise its structure, the gel was compressed with plastic after it had been incubated for one minute using a weight of 120 g. The gel was separated into homogeneous segments according to the particular samples to be treated once correctly aligned and stabilised. The gel segments were transferred to a 24-well plate, and each segment was seeded with a population of 1 × 10^5^ PC12 cells to create co-cultures. This crucial stage allows neurite expansion across the horizontal plane following the Schwann gels. The gel-containing 24-well plate was incubated for one hour at 37 °C. The process of neural cell adhesion to the collagen gel was aided by this incubation period.

### 2.8. MTT Viability

In accordance with the literature [26], the MTT-based In Vitro Toxicology Assay Kit (Merck Life Science, Rome, Italy) was used in all cell types. After stimulation, the cells were treated with 1% MTT dye for 2 h at 37 °C in a 5% CO_2_ and 95% humidity incubator. Analogous volumes of MTT Solubilization Solution were utilised to dissolve the purple formazan crystals. Optical equipment (Infinite 200 Pro MPlex, Tecan, Mannedorf, Switzerland) was employed to determine cell viability via absorbance measurement at 570 nm with correction at 690 nm. The outcomes were expressed as the means (%) SD of five separate experiments run in triplicate, and they were compared to the control (untreated samples, defined as the 0% line).

### 2.9. ROS Production

Using a conventional methodology [38], superoxide anion release was quantified by measuring cytochrome C reduction in culture supernatants at 550 nm (Infinite 200 Pro MPlex, Tecan, Männedorf, Switzerland). The O_2_ rate was reported as the mean SD (%) of nanomoles per decreased cytochrome C per microgram of protein, compared to the control value (0 line).

### 2.10. TNFα Assay

The TNFα concentration in the supernatants of HepG2, CCF-STTG1, and RSC96/PC12 cultures was determined using the TNF-α ELISA reagent (Merck Life Science, Rome, Italy) following the manufacturer’s instructions [48]. The absorbance was detected after adding a stop solution measurement plate at 340 nm (Infinite 200 Pro MPlex, Tecan, Mannedorf, Switzerland). The outcomes were represented as a mean (%) ± SD against the control (0% line) of five separate tests carried out in triplicate and compared to the standard curve (range from 93.75–6000 pg/mL of standard TNFα).

### 2.11. NFkB Analysis

Utilising the NF-kB (p65) Transcriptional factor Assay kit (Cayman Chemical Company, Ann Arbour, MI, USA) following the manufacturer’s instructions, NF-κB DNA binding activity was assessed on HepG2 cell lysates [49]. The absorbance at 450 nm was quantified utilising a spectrophotometer (Infinite 200 Pro MPlex, Tecan, Mannedorf, Switzerland). The obtained data were then compared to the standard curve produced by the NF-kB (p65) transcriptional factor positive control, which varied in concentration from 0 to 10µL/well based on dilutions that were scaled differentially. The findings were presented as the mean SD (%) of five separate experiments carried out in triplicate compared to the control (0 line).

### 2.12. Interleukin 1β Assay

The IL-1β ELISA kit (R&D systems, MN) was used to evaluate the culture supernatants of HepG2, CCF-STTG1, and RSC96/PC12 cells, following the instructions provided by the manufacturer [50]. The samples’ OD was compared to a standard curve (range from 3.906 to 250 pg/mL) using a plate reader (Infinite 200 Pro MPlex, Tecan, Männedorf, Switzerland) set to read the plate at 450 nm with correction at 570 nm. Five independent experiments were carried out in triplicate, and the results were presented as mean SD (%) versus control (0% line).

### 2.13. CYP1A2 Assay

CYP1A2 in HepG2 cells was quantified using the Human CYP1A2 ELISA Kit (Lifespan Biosciences, Lynnwood, Washington, United States) according to the manufacturer’s instructions. After incubating 100 µL of each lysate at 37 °C for 90 min, 100 µL of Biotinylated Detection Antibody was added and incubated for 1 h. After adding 100µL of HRP conjugate and incubating at 37 °C for 30 min, the mixture was rinsed thrice. The TMB substrate was incubated at 37 °C for 15 min using 90 µL. The plate was finally scanned at 450 nm using a plate reader (Infinite 200 Pro MPlex, Tecan, Männedorf, Switzerland) after 50 μL of stop solution was added. The outcomes were represented as mean SD (%) vs. control (0 line) of five independent tests carried out in triplicate and compared to a standard curve (range from 0.156 to 10 ng/mL).

### 2.14. CYP3A4 Assay

The Human CYP3A4 Cytochrome P450 3A4 ELISA Kit (Lifespan Biosciences, Lynnwood, WA, USA) was employed, as per the manufacturer’s instructions, to measure the amount of CYP3A4 in HepG2 cell lysate. Following an hour of incubation at 37 °C for 100 µL of each sample, 100 µL of Detection Reagent A was added and incubated for an additional hour at 37 °C. After exposing the plate to 100 µL of Detection Reagent B for 20 min at 37 °C, 90 μL of TMB substrate was added. Finally, 50 μL of stop solution was applied. An Infinite 200 Pro MPlex plate reader from Tecan, Männedorf, Switzerland, was used to read the plate at 450 nm. The data were then compared to a reference curve ranging from 0.313 to 20 ng/mL. The results of five triplicate independent experiments were presented as the mean standard deviation (%) relative to the control (0 line).

### 2.15. Src Activity

The Human Phospho-Src (Y419) Duo set ELISA Kit (R&D Systems, Minneapolis, MN, USA) was used to measure phospho-Src in HepG2 cell lysate according to the manufacturer’s instructions. The plate was incubated overnight at room temperature after adding the antibody at a concentration of 4.0 µg/mL and a volume of 100 µL. After cleaning the plate for two hours with wash buffer, 100 µL of each sample was added to each well, and the plate was then allowed to incubate at room temperature for an additional two hours. Following two hours at room temperature, 100 µL of the diluted detection antibody was added to each well. Streptavidin-HRP A was added, and the mixture was left for 20 min before being cleaned with a wash buffer. Lastly, each well received 100 μL of substrate solution, left in the dark for 20 min until 50 µL of stop solution was added. Using a plate reader (Infinite 200 Pro MPlex, Tecan, Männedorf, Switzerland), the OD was read at 570 nm. The findings were then normalised to the standard curve, which ranged from 3.9 to 250 pg/mL. Compared to the control (0 line), the outcomes of five independent experiments conducted in triplicate were presented as the mean standard deviation (%).

### 2.16. ERK/MAPK Activity

The ERK/MAPK activity in PNS lysates was assessed using the InstantOneTM ELISA (Thermo Fisher, Milan, Italy) according to the manufacturer’s instructions [51]. The ELISA strips were incubated at room temperature with shaking for one hour after adding 50 µL of each lysate. Stop solution was added after 20 min to stop the reaction before reading at 450 nm with a spectrometer (Infinite 200 Pro MPlex, Tecan, Männedorf, Switzerland). The outcomes were expressed as the average absorbance (%) relative to the control.

### 2.17. Amyloid Beta A4 ELISA Kit

The APP levels were measured on CCF-STTG1 supernatants using the Amyloid Beta A4 protein ELISA kit (Merck Life Science, Rome, Italy) by the ELISA kit HRP-conjugated manufacturer’s instructions [26]. APP concentration was evaluated by spectrometer absorbance at 450 nm (Infinite 200 Pro MPlex, Tecan, Männedorf, Switzerland). The data were quantified by comparing the results to the APP standard curve, which spans from 0.1 to 100 ng/mL. The values were then adjusted to the control values, represented by the 0 line.

### 2.18. pTAU Assay

The pTAU protein on CCF-STTG1 cell lysates was detected using the Tau (Phospho) [pS199] Human ELISA Kit (Thermo ScientificTM, Waltham, MA, USA), according to the manufacturer’s instructions, as documented in the literature [52]. Following the incubation of 100 μL of each sample with 100 μL of Detection Solution A, 100 μL of Detection Solution B, 90 μL of substrate solution, and 50 μL of stop solution, a spectrometer (Infinite 200 Pro MPlex, Tecan, Männedorf, Switzerland) was used to measure the absorbance at 450 nm. The data, expressed in ng/mL, were acquired by comparing them to the standard curve, which ranges from 15.6 to 1000 pg/mL. The mean SD (%) was then compared with the control (line 0).

### 2.19. p75 Activity by NGFR ELISA Assay Kit

The activity of p75 was measured using the Rat NGFR ELISA kit (MyBiosource, San Diego, CA, USA) according to the manufacturer’s instructions [38]. Each sample was aliquoted with 100 µL and incubated on the plate for 2 h at 37 °C. A measure of 100 µL of biotin antibody was added to the designated wells, and the plate was then incubated at a temperature of 37 °C for 60 min. Wash buffer was used to remove the solution from each well before washing it. A measure of 100 µL of HRP–avidin solution C was added to each well and allowed to sit at 37 °C for one hour. Each well was then filled with 90 µL of TMB substrate. Lastly, 50 µL of stop solution was added, and each well was left for 30 min at 37 °C. A spectrometer (Tecan Infinite 200 Pro MPlex, Tecan, Männedorf, Switzerland) was used to read the plates at 450 nm. The results ranged from 0.312 to 20 ng/mL and were collected and examined against the standard curve.

### 2.20. Myelin Protein Zero Detection

The MPZ level was investigated in cell lysates using the Myelin protein zero (MPZ) HRP-ELISA kit (MyBiosource, San Diego, CA, USA) according to the manufacturer’s instructions [38]. The concentration (ng/mL) was determined using a standard curve (range from 0.06 to 18 ng/mL) by a spectrometer (Infinite 200 Pro MPlex, Tecan, Männedorf, Switzerland), reading the plate at 450 nm. Compared to the control (0 line), the outcomes were presented as the means, with standard deviation (%), of the five independent experiments conducted in triplicate.

### 2.21. NRG1 Assay

The NRG1 Rat ELISA Kit (FineTest, Wuhan, China) used cell culture supernatants according to the manufacturer’s instructions. In summary, 100 µL of each sample was added to each well before the plate was incubated at 37 °C for 90 min. Upon the conclusion of the incubation time, the material within each well was extracted, and the wells were twice cleaned using a wash buffer. After adding 100 µL of the working solution containing biotin-labelled antibody into the wells mentioned above, the plate was incubated at a temperature of 37 °C for 6 min. After removing the solution from each well after incubation, each well was rinsed with a wash buffer three times. The wells were filled with 100 µL of SABC working solution, and the plate was incubated at 37 °C for 30 min. After five washing rounds, 90 µL of TMB substrate was applied to each well. A measure of 50 µL of stop solution was added to each well, and after 10 to 20 min, the plate was quickly inspected at 450 nm using a plate reader (Infinite 200 Pro MPlex, Tecan, Mannedorf, Switzerland). A standard curve with 0.156 to 10 ng/mL concentration ranges was used to compare the obtained data. The results of the five separate assays, each carried out in triplicate, were displayed as mean and standard deviation (%) compared to the control (0 lines).

### 2.22. Oestrogen Receptor Beta Activity

The Rat Oestrogen Receptor Beta (ERb) ELISA kit from Cloud-Clone in Houston, TX, USA, was employed to analyse cell lysates, following the guidelines provided by the supplier [53]. The spectrometer Infinite 200 Pro MPlex, Tecan, was used to measure the plate’s absorbance at 450 nm immediately after adding 50 µL of stop solution. Five independent experiments were conducted in triplicate, and the data obtained from the standard curve (which ranged from 0.312 to 20 ng/mL) were reported as the mean ± SD (%) vs. control.

### 2.23. Statistical Analysis

The findings provided in this study were obtained from a minimum of five separate experiments conducted in triplicate. The data were subsequently analysed via the Prism GraphPad statistical program. The results are reported as means ± standard deviation (%) and were analysed using one-way analysis of variance (ANOVA) followed by the Bonferroni post hoc test for statistical analysis. *p* < 0.05 was used to evaluate statistical significance.

## 3. Results

### 3.1. Cell Viability of NAC, VitD3, and Glut on CaCo-2 Cells

**Figure 2:**
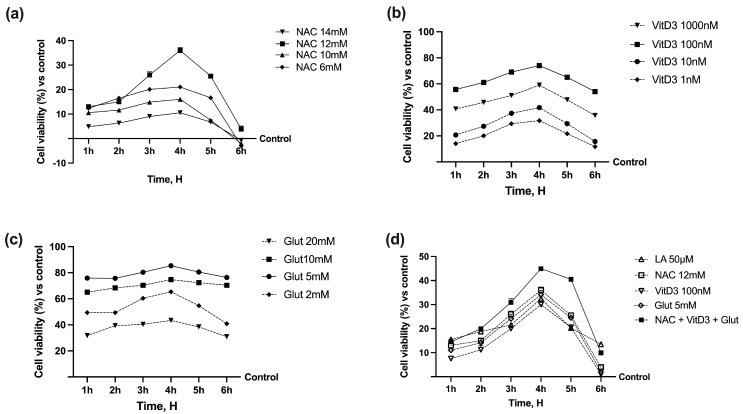
Dose–response and time-course study on cell viability of CaCo-2 cells. (**a**) In NAC; (**b**) VitD3; (**c**) Glut tested alone by MTT test from 1 to 6 h. (**d**), the same agents were added in combination compared to LA 50 µM. Data are expressed as mean (%) ± SD of five independent experiments performed in triplicates vs. control values (0% line).

The dose–response effect of NAC (ranging from 6 mM to 14 mM) on cell viability in a time-course study (from 1 h to 5 h) was examined in CaCo-2 cells. The viability revealed the time- and concentration-dependent effects of NAC compared to the control (*p* < 0.05), and all concentrations tested maintained their positive influence over time, excluding cytotoxicity, as reported in Figure 2a. NAC 12 mM displayed increased viability better than the other concentrations examined (*p* < 0.05), which is why it was maintained in all subsequent experiments. At the same time, further tests were performed to confirm the correct concentration of Glut and VitD3 to combine with NAC. As reported in Figure 2b, VitD3 enhanced the vitality of CaCo-2 cells in time- and dose-dependent effects compared to the control (*p* < 0.05), with a peak of viability around 4 h of stimulations with all concentrations tested. In detail, 100 nM VitD3 had the greatest trend (*p* < 0.05 vs. control), indicating that this dose may be the most effective among the various concentrations examined. In addition, as evidenced in Figure 2c, 5 mM Glut appears to have the best impact compared to the control (*p* < 0.05) with a peak of around 4 h of stimulation as the other concentrations tested. Based on the results obtained, the research was conducted in the following intestinal barrier investigations to assess the effects of NAC 12 mM in combination with VitD3 100 nM and Glut 5 mM. As shown in Figure 2d, the combination of NAC + VitD3 + Glut significantly increased cell viability compared to the control (approximately 46% vs. control, *p* < 0.05 at 4 h). These results led to the hypothesis that NAC can collaborate with VitD3 and Glut to maintain cell homeostasis and enhance intestinal cell activity. In addition, the combined effect was higher than LA 50 µM (*p* < 0.05 at 4 h, about 28%), supporting the hypothesis that the combination may be used as an alternative to it.

### 3.2. NAC, VitD3, and Glut Permeability and Absorption Analysis through Intestinal Barrier

Subsequent studies were conducted to investigate the absorption rate, as demonstrated in Figure 2, as all chemicals tested impacted the time-dependent intestinal barrier. The effect peaked at 4 h compared to the control and other time points (*p* < 0.05). The combination of NAC 12 mM, VitD3 100 nM, and Glut 5 mM exhibited a more physiological absorption rate (*p* < 0.05) than the individual components. This combination also preserved epithelial integrity and increased the flow of paracellular exchange ions through the intestinal epithelium. This combination effect seems to be better than LA 50 µM (*p* < 0.05, about 56%), indicating a possible improvement of epithelia during the consumption of the agents.

**Figure 3:**
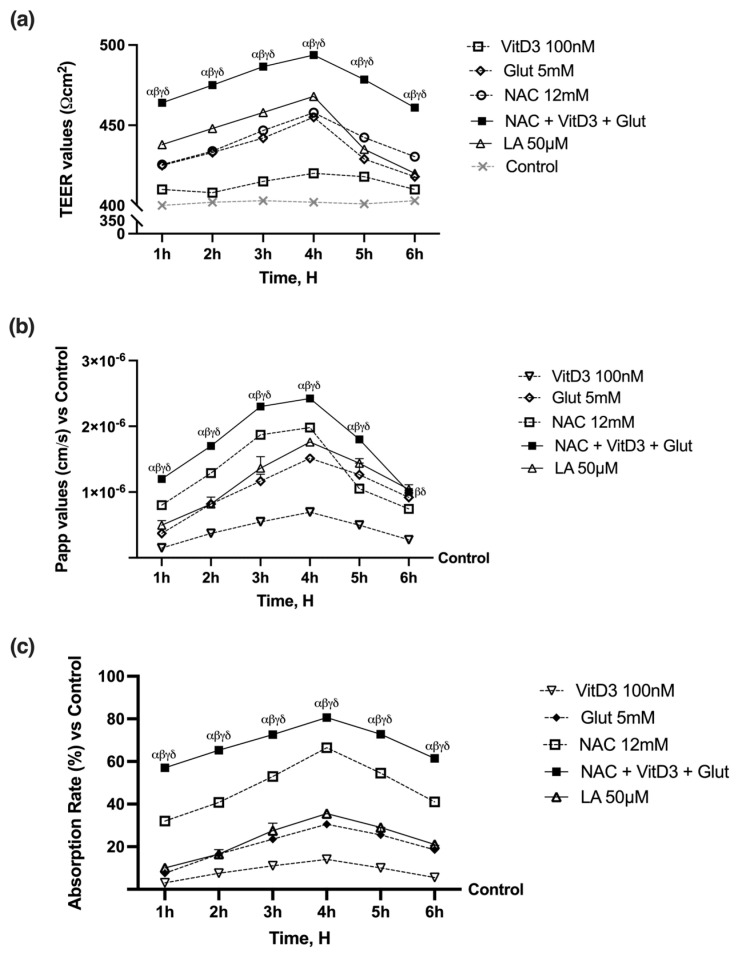
Intestinal activity on 3D in vitro model of the intestinal barrier. In (**a**), the TEER value is determined using the EVOM3 device. In (**b**), the Papp values are calculated as mentioned earlier. In (**c**), the passage through the intestinal barrier is evaluated using a fluorescent tracer. The data are reported as means ± SD (%) of five independent experiments performed in triplicate and normalized to control values (the 0% line). α *p* < 0.05 vs. Lipoic Acid 50 µM; β *p* < 0.05 vs. VitD3; γ *p* < 0.05 vs. glutathione; δ *p* < 0.05 vs. NAC 12 mM.

The analysis of TEER values supports the cell viability previously observed, excluding cytotoxic effects due to the stimulation with all agents. Figure 3a,b demonstrates how combining NAC with Glut and VitD3 increased the ion flow of paracellular exchanges through the intestinal epithelium with a better effect than the single agents (*p* < 0.05). According to the permeability study (Figure 3b) NAC + VitD3 + Glut produced the greatest results compared to the control (*p* < 0.05). Comparing the data to the control group and the corresponding single agents revealed a similar trend with NAC + VitD3 + Glut (*p* < 0.05), supporting that NAC, VitD3, and Glut work together to improve intestinal activity. NAC + VitD3 + Glut also produced the greatest results, increasing intestinal barrier permeability at 4 h compared to LA 50 µM (*p* < 0.05), indicating that the utilized combination had a dose-dependent impact. The trend found by the analysis of the Papp was further confirmed by the absorption analysis (Figure 3c). Specifically, the data demonstrated that NAC + VitD3 + Glut was more bioavailable (*p* < 0.05) when compared to each agent alone and LA 50 µM (*p* < 0.05), demonstrating that a large amount of the tested compounds crosses through the intestinal barrier and reach the circulation. The basolateral environment of each Transwell^®^ insert was collected and used to stimulate the hepatic cells.

### 3.3. NAC, VitD3, and Glut Role in the Hepatic Cell Line

**Figure 4:**
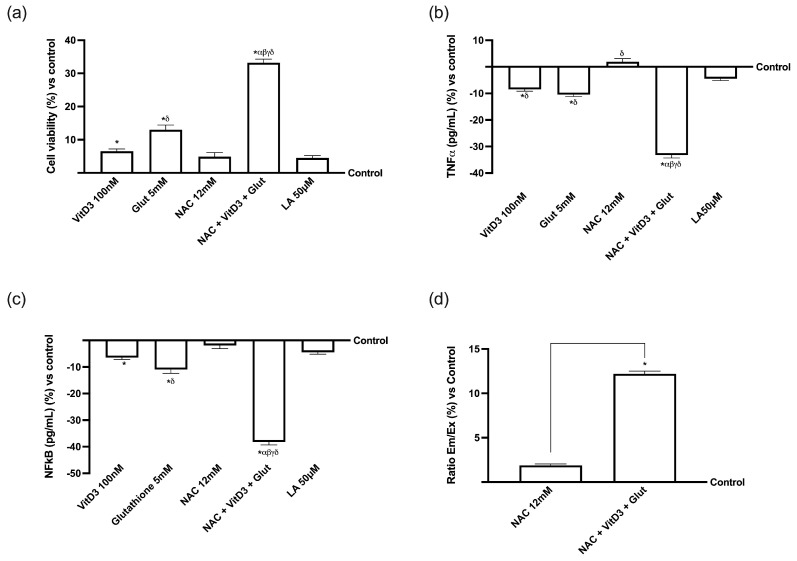
Hepatic activity on the 3D in vitro model stimulated with the combination of NAC 12 mM in the presence of VitD3 100 nM and Glutathione 5 mM and compared to LA 50 µM. (**a**) Cell viability measured by MTT test; (**b**) TNFα measurement by ELISA kit; (**c**) NF-Kb measurement by ELISA kit; (**d**) NAC dosage performed with spectrophotometry technique. Data are expressed as means ± SD (%) of five independent experiments performed in triplicate and normalized to control values (the 0% line). In (**a**–**c**), * *p* < 0.05 vs. control; α *p* < 0.05 vs. Lipoic Acid 50 µM; β *p* < 0.05 vs. VitD3; γ *p* < 0.05 vs. glutathione; δ *p* < 0.05 vs. NAC 12 mM. In (**d**), * *p* < 0.05 vs. control and the bars *p* < 0.05 vs. NAC 12 mM.

The effects of NAC 12 mM, in combination with VitD3 100 nM and Glut 5 mM, were assessed on a liver 3D model after passage through the intestinal barrier to determine the alteration of biochemical processes, excluding cytotoxicity, the amount of substances that cross the liver, and the inflammatory response. At the same time, the same analysis was also performed in the presence of LA 50 µM. HepG2 cells were treated for 24 h with media obtained from the intestinal model’s basolateral compartment after being stimulated for 4 h. As shown in Figure 4a, the viability of liver cells was significantly enhanced by each tested substance after 24 h of treatment, compared to the control group (*p* < 0.05). Specifically, NAC is taken up by the hepatic cells (*p* < 0.05 vs. control). This effect was amplified by the combination of NAC + VitD3 + Glut, which increased the viable cells (about 14% compared to NAC alone, *p* < 0.05); this effect was greater than LA50 µM (about 85%, *p* < 0.05), supporting the important role of the combination to act in similar ways to LA 50 µM. Similar results were also observed analysing both TNFα and NF-kB (Figure 4b,c) that revealed the greater effects of the combination composed of NAC + VitD3 + Glut (approximately 86% and 88%, respectively, *p* < 0.05). Additionally, the extracellular/intracellular ratio of the NAC-crossed hepatic tract was quantified (Figure 4d); in comparison to the control and after a single dosage, the examination revealed an increase in the NAC absorption capacity (*p* < 0.05). In the permeability experiment, NAC + VitD3 + Glut had a more appreciable absorption than NAC alone (about 84%, *p* < 0.05). These results further prove that the combinations’ ability to traverse the intestinal barrier and penetrate the hepatic environment may result in beneficial outcomes.

**Figure 5:**
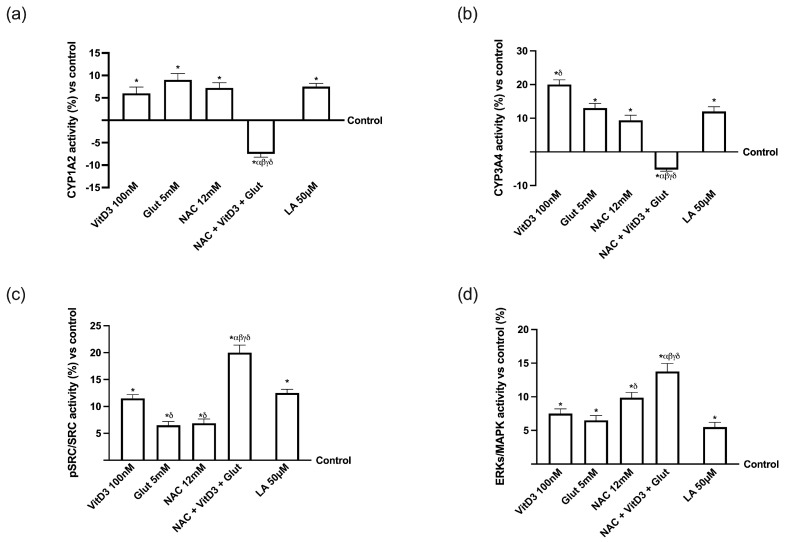
Analysis of intracellular markers involved in hepatic homeostasis on a 3D in vitro liver model after 24 h. (**a**) CYP1A2, (**b**) CYP3A4, (**c**) SRC, and (**d**) ERK/MAPK activities measured by ELISA kit. Data are mean ± SD of five independent experiments performed in triplicates and expressed as percentages (%) vs. control values (0% line). * *p* < 0.05 vs. control; α *p* < 0.05 vs. Lipoic Acid 50 µM; β *p* < 0.05 vs. VitD3; γ *p* < 0.05 vs. glutathione; δ *p* < 0.05 vs. NAC 12 mM.

To evaluate the hepatic metabolism of the substances tested, the main Cyp450 isoforms, Cyp1A2 and Cyp3A4, as well as Src and extracellular signal-regulated kinase/mitogen-activated protein kinase (ERK/MAPK), were also examined. As shown in Figure 5a,b, the proper mechanisms of Cyp1A2 and Cyp3A4 are activated by NAC 12 mM alone but not when it is both coupled with VitD 100 nM and Glut 5 mM (*p* < 0.05), matching with the results previously obtained on hepatic metabolism. Additionally, identical findings were found when analysing Src and ERK/MAPK (Figure 5c,d); NAC + VitD3 + Glut controlled the activities of both parameters better than the single agents tested (*p* < 0.05), which led to the assumption that these combinations can improve the liver cells metabolism. These findings confirm that NAC, in combination with VitD3 and Glut, can keep the liver in a homeostatic state, and these results were similar to what was observed by LA 50 µM, supporting the hypothesis to substitute LA with a new combination. To create the subsequent in vitro model, the basolateral environment of each Transwell^®^ support stimulation was collected.

### 3.4. Beneficial Effects of NAC, VitD3, and Glut on Astrocytes Crossing the BBB

**Figure 6:**
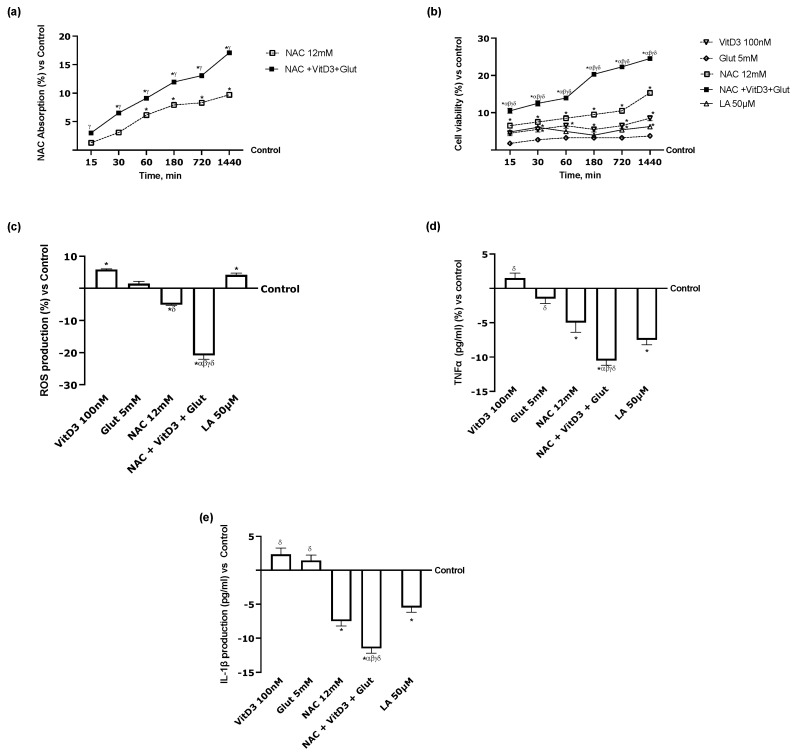
Analysis of the beneficial effects on CCF-STTG1 cells. (**a**) NAC quantification by spectrophotometry technique and (**b**) cell viability measured by MTT test in a study from 1 h to 6 h; (**c**) ROS production analysis measured through cytochrome C reduction in CCFTG cells after 24 h; (**d**) TNFα and (**e**) IL1-*β* activities measured by ELISA kit on CCFTG cells after 24 h. Data are mean ± SD of five independent experiments performed in triplicates vs. control values (0% line). * *p* < 0.05 vs. control; α *p* < 0.05 vs. Lipoic Acid 50 µM; β *p* < 0.05 vs. VitD3; γ *p* < 0.05 vs. glutathione; δ *p* < 0.05 vs. NAC 12 mM.

Additional tests were carried out to determine whether VitD3 and Glut could cross the BBB and positively influence neuronal cells, to assess the effectiveness of NAC in combination with these two compounds. For this purpose, the BBB was treated with the medium taken from the basolateral environment of the 3D liver in vitro model for 24 h, specifically from 15 min to 1440 min; NAC absorption was assessed after each time point. Figure 6a shows that, compared with the control, NAC uptake increased, starting at 15 and continuing to 1440 min. These results confirm the value of employing NAC 12 mM in combination with VitD3, Glut, and other compounds to promote greater absorption under physiological settings. To comprehend how effectively NAC may reach the CNS and shield it from harm when paired with VitD3 and Glut, astrocyte cell viability research was carried out. Figure 6b demonstrates that the study revealed a time-dependent increase in cell viability caused by NAC 12 mM alone compared to the control (*p* < 0.05). The addition of VitD3 and Glut increased NAC activity time-dependently (*p* < 0.05). Indeed, NAC + VitD3 + Glut demonstrated a significant effect compared to the single agents through all the time ranges tested (*p* < 0.05). These results confirm that NAC + VitD3 + Glut can lower neuronal susceptibility and may be a possible alternative for LA 50 µM, which exerts a lower effect than the combination (*p* < 0.05). In addition, ROS production analysis (Figure 6c) revealed a similar trend, demonstrating once again the positive effect of the combination compared to the single agents (*p* < 0.05). Even in this case, NAC + VitD3 + Glut exerted a significant effect compared to LA 50 µM, reducing ROS production by 1.2 times more (*p* < 0.05). These results were further supported by the analysis of the inflammatory response (Figure 6d,e), which shows that NAC 12 mM significantly reduces the production of proinflammatory cytokines when compared to the control (*p* < 0.05). NAC + VitD3 + Glut had a stronger effect on both parameters examined than the single agents (*p* < 0.05). All these findings align with the hypothesis that NAC, VitD3, and Glut may directly affect astrocyte survival due to their ability to traverse the BBB even better than LA 50 µM.

**Figure 7:**
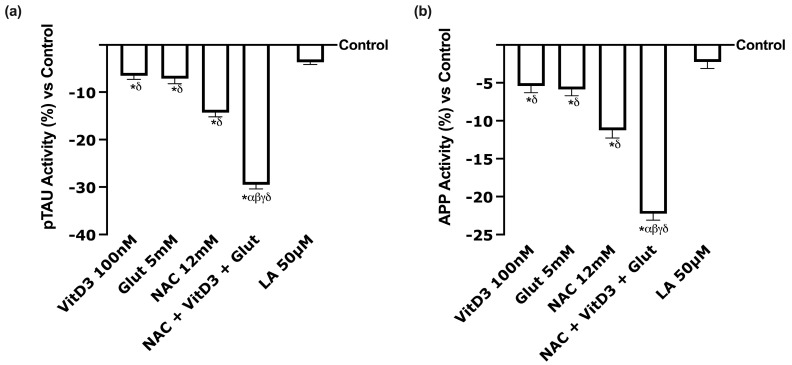
Analysis of biomarkers in CCF-STTG1 cells. (**a**) pTAU activity and (**b**) APP activity measured by ELISA kit on CCFTG cells after 24 h. Data are mean ± SD of five independent experiments performed in triplicates vs. control values (0% line). * *p* < 0.05 vs. control; α *p* < 0.05 vs. Lipoic Acid 50 µM; β *p* < 0.05 vs. VitD3; γ *p* < 0.05 vs. glutathione; δ *p* < 0.05 vs. NAC 12 mM.

To further investigate the beneficial effects of NAC, VitD3, and Glut compared to LA, pTAU and APP activity were also analysed to determine the specific markers associated with brain alterations. As observed in Figure 7a, all the single agents, excluding LA, can induce a significant effect compared to the control (*p* < 0.05); specifically, the better effect was induced by NAC and its combination with VitD3, and Glut improved this impact. Indeed, NAC + VitD3 + Glut showed a significant effect compared to all its single agents and LA 50 µM (*p* < 0.05, approximately 87%). Similar results were obtained also investigating APP activity (Figure 7b). In detail, NAC + VitD3 + Glut not only demonstrated a significant effect compared to all its single agents (*p* < 0.05) but even compared to LA 50 µM (*p* < 0.05, approximatively 90%). These data demonstrated the beneficial effect of NAC, VitD3, and Glut at the CNS level.

**Figure 8:**
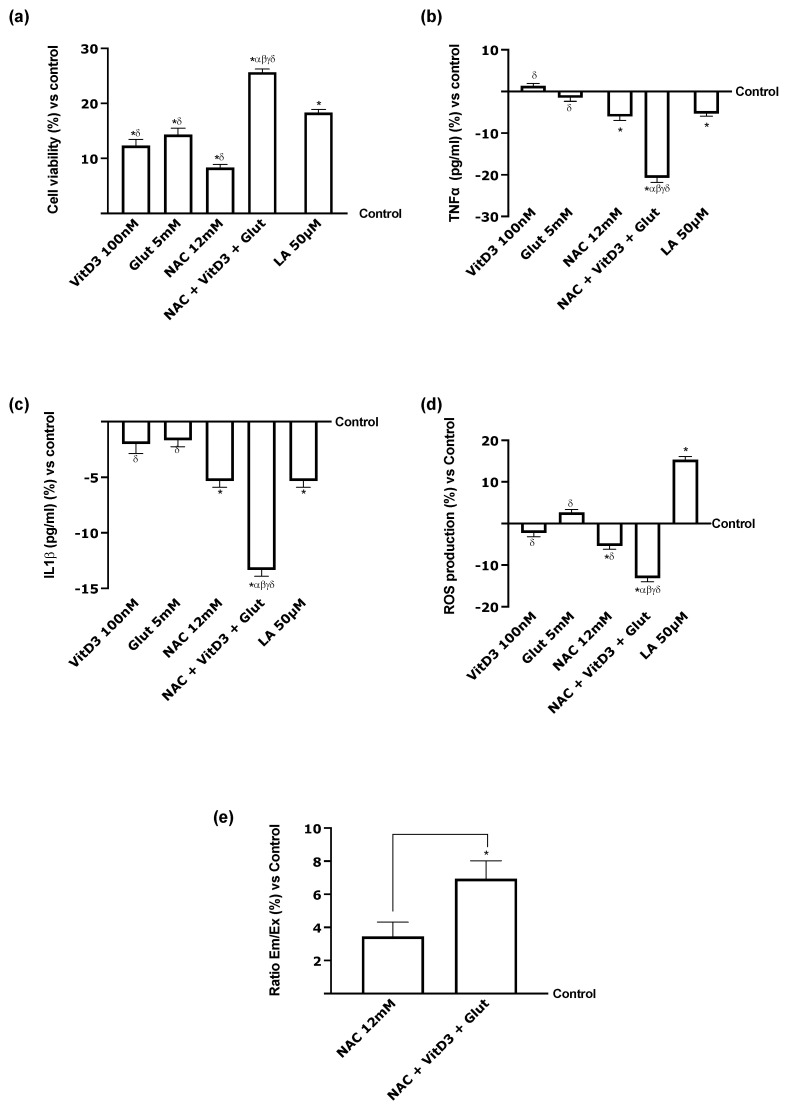
Effects of the substances on PNS in vitro model after 24 h. (**a**) Cell viability measured by the MTT test; (**b**) TNFα and (**c**) IL1-*β* activities analysed by ELISA kit; (**d**) p75 activity performed by ELISA kit; (**e**) NAC concentration determined by spectrophotometry technique. In all panels, VitD3 100 nM and Glut 5 mM are also reported. Data are mean ± SD of five independent experiments performed in triplicates vs. control values (0% line). * *p* < 0.05 vs. control; α *p* < 0.05 vs. Lipoic Acid 50 µM; β *p* < 0.05 vs. VitD3; γ *p* < 0.05 vs. glutathione; δ *p* < 0.05 vs. NAC 12 mM.

Further tests were carried out using a co-culture model made up of RSC-96 and PC12 cells since the effects of NAC coupled with VitD3 and Glut might be considered an effective method for preserving the health of peripheral nerve tissue. Figure 8 demonstrates that, following hepatic transit, all substances tested may reach the target without deleterious impacting cell survival or inflammatory response (*p* < 0.05). Particularly, NAC + VitD3 + Glut showed a very significant impact on the preservation of mitochondrial health and, concurrently, on the reduction in the inflammatory process, as determined by TNFα and IL-1 productions and oxidative stress by a reduction in ROS production (Figure 8a–d, *p* < 0.05). All of these results supported the use of the combination composed of NAC + VitD3 + Glut to produce more significant effects compared to LA 50 µM (*p* < 0.05) on all parameters analysed, probably due to its ability to reach better the target organs (Figure 8e).

**Figure 9:**
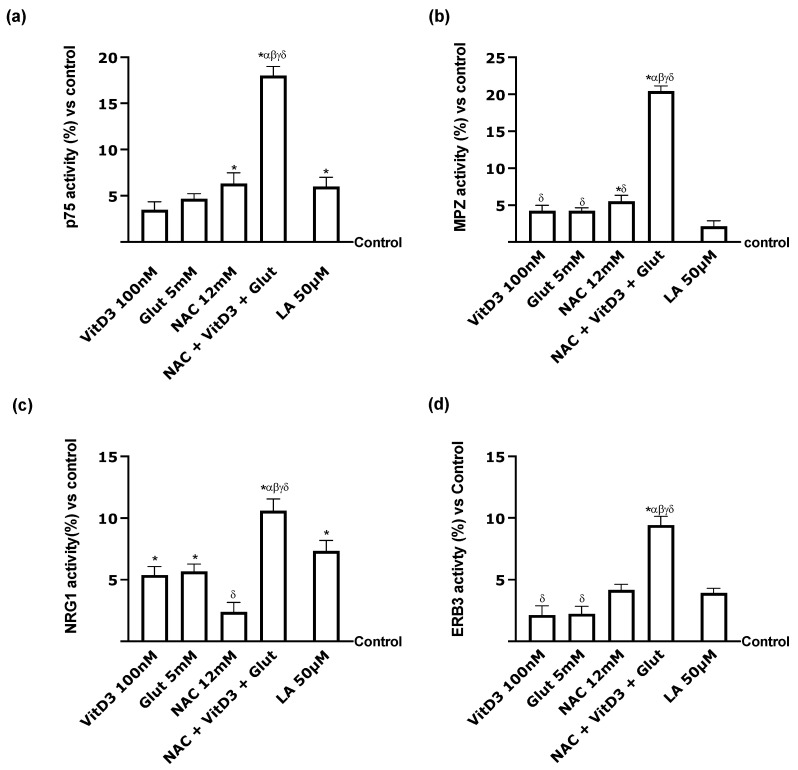
Analysis of Schwann cell activity after 24 h. (**a**) p75; (**b**) MPZ; (**c**) NRG1; (**d**) ERB3 activities analysed by ELISA kit. Data are mean ± SD of five independent experiments performed in triplicates vs. control values (0% line). * *p* < 0.05 vs. control; α *p* < 0.05 vs. Lipoic Acid 50 µM; β *p* < 0.05 vs. VitD3; γ *p* < 0.05 vs. glutathione; δ *p* < 0.05 vs. NAC 12 mM.

Finally, showing that the combination may enter the PNS while still having positive effects is critical. Further tests were carried out to understand better the neurotrophic receptor, p75 (Figure 9a). Specifically, NAC + VitD3 + Glut increased p75 activity compared to the single components (*p* < 0.05) but also compared to LA 50 µM by approximately 67% (*p* < 0.05), demonstrating once again the better activity of the combination. The same framework was observed for MPZ activity analysis (Figure 9b); however, in this case, all the single agents of the combination exerted a significant effect even compared to LA 50 µM (*p* < 0.05). This effect was further induced by NAC + VitD3 + Glut, which increased MPZ activity by approximately 90% compared to LA 50 µM (*p* < 0.05). Finally, a similar trend was observed for NRG1 and ERB3 activity (Figure 9c,d), supporting the hypothesis that NAC + VitD3 + Glut can improve nerve injury and restore the myelination process. These investigations were crucial to understanding the effectiveness of NAC, VitD3, and Glut in preserving PNS physiological homeostasis to introduce their use as an alternative strategy to LA50 µM, which has a similar mechanism of action.

## 4. Discussion

In recent years, the importance of developing approaches to precisely assess foods’ antioxidant activity and capacity and their components within the entire organism has increased [54]. Specifically, over 10,000 molecules with anti-inflammatory, immunomodulatory, and antimutagenic activities have been discovered so far, and their biological function is mostly determined by their chemical structure [55]. Indeed, antioxidants are necessary to inhibit the generation of new free radicals to stop oxidative stress, and the administration of exogenous antioxidant chemicals can prevent ROSs from exerting their harmful effects [55]. For instance, several studies have reported the antioxidant effects of NAC [13,56], VitD3 [57,58], and Glut [59,60], highlighting their significant contributions to enhancing the mechanisms responsible for maintaining body homeostasis and safeguarding the health of body tissues. The data collected in this study demonstrate that the combination of NAC, VitD3, and Glut exerts a cooperative effect in all body districts analysed, indicating a possible new strategy to restore oxidative homeostasis of the body. Thus, an intriguing dietary supplementation method would be to use NAC, VitD3, and Glut as alternative therapeutic agents to Lipoic Acid, as summarized in the diagram (Appendix A). The current in vitro research was conducted to accomplish this goal by joining various cell lines to simulate the route a food supplement takes through the body when taken orally, to reach the target organ. Using this method, the data collected exhibit the role of NAC, VitD3, and Glut’s combination in exerting beneficial effects after crossing the intestinal barrier. The findings showed that NAC, VitD3, and Glut cooperate in all body regions studied, suggesting a potentially novel approach to reestablishing the body’s oxidative homeostasis. According to that, the NAC concentration exerted a stronger dose-dependent beneficial activity. In particular, NAC 12 mM showed the largest effects in all compartments compared to other NAC dosages. The most effective NAC dosage to employ, combined with VitD3 100 nM and Glut 5 mM, since they can enhance intestinal cell viability, is 12 mM, according to our results from a preliminary dose–response study to determine the minimum effective concentration on CaCo-2 cells. Considering this, our research started by finding each functional compound’s positive effects, either alone or in combination, strongly related to its intestinal absorption. The degree to which nutrients or bioactive principles present in foods are absorbed into the bloodstream and redistributed in the body was evaluated. As a result, both permeability through the intestinal barrier and the subsequent bioavailability were assessed. These traits pose a significant challenge in developing nutraceuticals [61]. Therefore, we can confidently state that the combination of NAC + VitD3 + Glut has proven to be highly effective along the intestinal tract, without causing any harmful effects on cells. This combination not only enhances the bioavailability of NAC but also increases the accessibility of various compounds for absorption. According to these facts, all compounds are absorbed without the possibility of low or variable absorption levels, changes in transport mechanisms, bioavailability, or bioactivity, which are essential for understanding. Thus, the high bioavailability of these natural antioxidants considerably increases their biological activity even at low doses, as demonstrated by this innovative formulation technique, which uses the combined effect of NAC, VitD3, and Glut.

Hence, the subsequent phase of this study involved conducting a thorough examination of the availability of the compound after it passes through the intestines, as well as studying its metabolism by HepG2 cells. Indeed, the liver is essential in metabolizing drugs taken orally and transported to the body’s target organs. This stage is crucial for simulating how a supplement is metabolized and distributed through the body following oral ingestion and intestinal absorption in vitro. Regarding the effectiveness of NAC as a therapeutic agent, it has been observed that it is hindered by certain limitations associated with its pharmacokinetic properties. Numerous studies have highlighted a relatively short duration of action and a bioavailability of approximately 6–10% [62]. The cellular and molecular mechanisms through which NAC + VitD3 + Glut affect liver metabolism have been shown to maintain normal hepatic homeostasis. Therefore, it is crucial to keep that in mind. Without having any adverse effects, this mixture increases the entry of NAC and other ingredients. It is important to note that NAC absorption in the liver may have affected how phase I and II metabolism were regulated, including the inhibition of cytochrome P450-dependent enzymes. In addition, a statistically significant reduction in CYP1A2 and CYP3A4 was observed after the administration of the NAC + VitD3 + Glut; our results may indicate the ability of the combination not to interfere with homeostatic liver metabolism and not to alter bioavailability, increase toxicity, or compromising therapeutic effectiveness. According to these data, we can conclude that the combination based on NAC, VitD3, and Glut benefits the body. The potential of NAC + VitD3 + Glut to easily cross the BBB after hepatic passage and produce favourable effects, as we have seen in both the CNS and PNS model, makes it another viable agent for enhancing the quality of life and maintaining good effects on PNS and CNS.

Furthermore, NAC, when combined with VitD3 and Glut, not only can alleviate the inflammatory response as shown by lowering the level of TNFα and either IL1*β* in CNS or IL-6 in PNS, but also has the potential to enhance the production of neurotrophins in the PNS. In particular, in the presence of the combination, the level of p75, a positive modulator for Schwann cell myelination, modestly increases compared to the control (approximately 18%). A similar trend was observed for NRG1 and ERB3 activity, pathways involved in the myelination of Schwann cells. In conclusion, the beneficial effects on the nervous system cells are enhanced because a significant amount of substance can reach both neurons and Schwann cells due to the combined cooperative effect of NAC, VitD3, and Glut. Indeed, NAC protects cells from oxidative stress and improves glutathione availability [63], and the presence of VitD3 supports the optimal mitochondrial function reduction in the inflammatory network due to its ability to induce genomic and non-genomic effects [18,64].

## 5. Conclusions

In conclusion, this study demonstrates for the first time that the combination of NAC, VitD3, and Glut has therapeutic efficacy without limitations related to the pharmacokinetic profile. In addition, the combined treatment with NAC 12 mM + VitD3 100 nM + Glut 5 mM spreads through the entire body after oral intake, without altering the homeostatic mechanisms of each compartment, while maintaining proper functionality; thus, it may be classified as a candidate formulation for the prevention or slowing down of some conditions associated with oxidative stress disease. However, more controlled and robust in vivo and clinical trials must be designed to investigate the therapeutic effects of NAC 12 mM + VitD3 100 nM + Glut 5 mM.

## Figures and Tables

**Figure A1:**
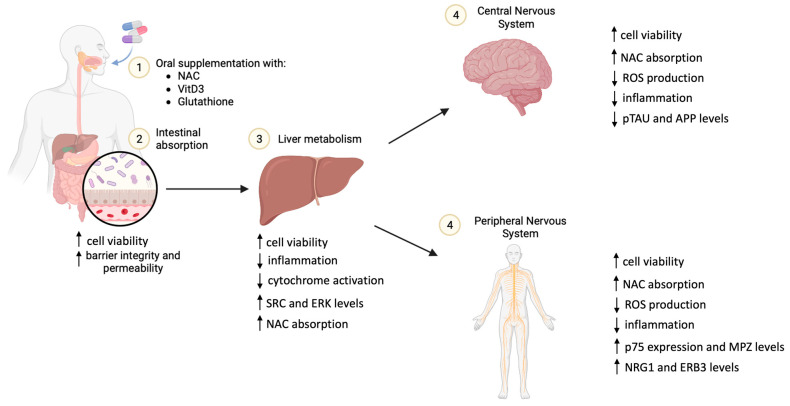
Summary of the main results obtained in each phase.

## Data Availability

The original contributions presented in the study are included in the article, further inquiries can be directed to the corresponding author.

## Abbreviations

ANOVA	one-way analysis of variance
ATCC	American Type Culture Collection
BBB	blood–brain barrier
CNS	central nervous system
DMEM-Adv	Dulbecco’s Modified Eagle’s Medium Advance
EGM-2	Endothelial Growth Medium-2
EMA	European Medicines Agency
ERK	extracellular signal-regulated kinase
FBS	Foetal Bovine Serum
FDA	Food and Drug Administration
GA-1000	gentamicin sulphate-amphotericin
hEGF	human epidermal growth factor
hFGF-B	human basic fibroblast growth factor
HS	horse serum
IL	Interleukin
MEM	minimum essential medium
MAPK	mitogen-activated protein kinase
MTT	3-(4,5-Dimethylthiazol-2-yl)-2,5-diphenyltetrazolium bromide
NAC	N-acetylcysteine
Papp	apparent permeability coefficient
PNS	peripheral nervous system
R3-IGF-1	insulin-like growth factor-I
ROS	reactive oxygen species
RPMI	Roswell Park Memorial Institute medium
TEER	transepithelial electrical resistance
TNFα	tumour necrosis factor alpha
VEGF	vascular endothelial growth factor
VitD3	vitamin D3
